# Protective Effect of Dietary Taurine from ROS Production in European Seabass under Conditions of Forced Swimming

**DOI:** 10.3390/ani9090607

**Published:** 2019-08-26

**Authors:** Chiara Ceccotti, Basim S.A. Al-Sulaivany, Omar A.M. Al-Habbib, Marco Saroglia, Simona Rimoldi, Genciana Terova

**Affiliations:** 1 Department of Biotechnology and Life Sciences, University of Insubria, 21100 Varese, Italy; 2 Department of Biology, Faculty of Science, University of Zakho, Zakho, 420011 Kurdistan Region, Iraq; 3 College of Science, International University of Erbil, 44001 Kurdistan Region, Iraq

**Keywords:** aquaculture, respiratory burst activity, critical swim speed, reactive oxygen species, feed additive

## Abstract

**Simple Summary:**

A number of recent studies have demonstrated the essentiality of dietary taurine in many commercially relevant cultured fish species. Taurine is involved in many physiological functions in fish and represents an essential nutrient, which exert powerful antioxidant properties and is required as a supplement in the feed when a relevant percentage of vegetable protein sources are utilized. Our results show that dietary taurine can reduce the oxidative status of marine fish under swim stamina stress conditions. These data could be essential for the development of diets with fishmeal/soybean meal substitutions in the effort to improve the sustainability of the aquaculture.

**Abstract:**

Taurine (Tau) is an amino sulfonic acid, which is widely distributed in animal tissues, whereas it is almost lacking in plants with the exception of certain algae, seaweeds, and few others. In the aquafeed industry, Tau is mainly used as a feed additive to promote growth in marine fish species with limited cysteine sulfinate decarboxylase activity. In particular, Tau supplementation is required in feeds in which fishmeal (FM) is substituted with high percentages of plant-derived protein sources such as soybean meals (SBM) that have much lower levels of Tau than FM. In addition to being a growth promoter, Tau exert powerful antioxidant properties being a scavenger of the reactive oxygen species (ROS). Under sustained swimming conditions, an intracellular increase in ROS production can occur in fish red muscle where the abundance of mitochondria (the main site of ROS formation) is high. Accordingly, this study aimed at investigating the effects of dietary Tau on European seabass (*Dicentrarchus labrax*) growth and oxidative stress response induced by swimming exercise. Individually tagged fish of 92.57 ± 20.33 g mean initial weight were fed two experimental diets containing the same low percentage of FM and high percentage of SBM. One diet was supplemented with 1.5% of Tau. Tau supplemented in the diet had a positive effect on fish growth, and enhanced swimming performance and antioxidant status. Two swim endurance tests were performed during the feeding trial. Metabolic oxygen consumption (MO_2_) was measured during exercise at incremental swimming speeds (0.7, 1.4, 2.1, 2.8, 3.5, and then 4.2 BL (body length) s^−1^, until fatigue). Fish maximal sustainable swimming speed (Ucrit) was determined too. To investigate the antioxidant effect of dietary Tau, we also measured ROS production in fish blood by RBA (respiratory burst activity) assay and quantified the expression of genes coding for antioxidant enzymes by qPCR (quantitative polymerase chain reaction) , such as SOD (superoxide dismutase), GPX (glutathione peroxidase), and CAT (catalase) in red muscle and liver. There was a significant effect of Tau upon Ucrit during exercise. Additionally, ROS production was significantly lower in fish fed with Tau supplemented diet, supporting the role of Tau as ROS scavenger. The protective effect of Tau against oxidative stress induced by forced swimming was denoted also by a significant decrease in antioxidant enzymes gene expression in fish liver and muscle. Taken together these results demonstrate that Tau is beneficial in low FM-based diets for seabass.

## 1. Introduction

Fishmeal (FM) supplies are declining and the aquaculture sector is urgently searching for alternative sources of protein. Terrestrial plant sources, such as soybean meal (SBM), are promising due to their high-protein content that satisfy nutritional requirements of carnivorous fish species. Indeed, SBM is the most common protein source used in aquafeeds due to its availability worldwide and consistent amino acid supply profile at low cost [1]. Although the use of SBM has given positive results in marine fish feeds, the presence of anti-nutritional factors, and the scarcity of indispensable amino acids such as methionine and lysine, has reduced fish performance. Another reason for the decrease in growth in fish fed plant-protein-based diets is the lack of the amino sulfonic acid taurine (Tau), which is an organic compound abundant in FM and other animal-based feeds [2]. Indeed, Tau is widely distributed in animal tissues, whereas it is almost lacking in plants with the exception of certain algae, seaweeds, and a few others, such as cactus pear.

In mammals, the beneficial effects of Tau have been widely investigated [3,4,5,6,7]. In the last few years, research in aquaculture has also focused on the effects of Tau on freshwater and marine fish species’ physiology, metabolism, nutrition, and culture [8]. Indeed, several studies have tested Tau used as a feed additive to improve the metabolic parameters of fish fed with low FM-based diets. Different fish species such as cobia [9], red sea bream [10], and European seabass [11] have shown a positive effect on growth performance as a result of Tau supplementation in the diet. However, the optimal growth and survival rates ascribed to dietary Tau have not been documented in all life stages of fish [2]. Such information would be valuable to refine requirement estimates and the necessary supplementation levels depending on fish life stages. Indeed, the growth stimulatory effects of dietary Tau have mainly been correlated with the development stages (ontogenesis) and fish size, and were strictly linked to the species [12]. 

The role of cysteine sulfinate decarboxylase (CSD) in fish is crucial for Tau synthesis. In marine fish, the activity of this enzyme in the liver is low or absent [13] and dietary supplementation of taurine is suggested in these fish species. For instance, in larval stages of several carnivorous marine fish, enriching live prey with Tau increased growth and survival parameters, and also promoted the morphological development and the activity of digestive enzymes [8]. In contrast, rainbow trout (*Oncorhynchus mykiss*), and Japanese flounder (*Paralichthys olivaceus*) showed high levels of CSD activity, but they were however lower than levels found in mammalian species [14]. Nevertheless, Tau supplementation essentially maximized growth, both in species with deficient CSD activity, and in those with a sufficient rate of Tau synthesis [14,15].

In addition to its role as a growth promoter, the nutraceutical Tau has several other properties, such as functioning as an anti-oxidizing agent, supporting osmoregulation, stabilizing and protecting cell membranes [4], and scavenging reactive oxygen species (ROS) [16]. 

ROS, such as superoxide radicals, hydrogen peroxide, and hydroxyl radicals, are all produced through sequential one-electron reduction of O_2_ that occurs in the mitochondria under physiological conditions. When intracellular homeostasis is altered and the balance between pro- and anti-oxidants cannot control the ROS level, the oxidative stress develops. As a consequence of this stress, ROS attack proteins, phospholipids, or DNA, promoting cell necrosis. However, to contrast ROS, different enzymes such as superoxide dismutase (SOD), catalase (CAT), and glutathione peroxidase (GPx) are produced by the cells to act as a first line of defense against the oxidative stress [17]. SODs remove damaging ROS from the cells by alternately catalyzing the partitioning of superoxide radical to either hydrogen peroxide or molecular O_2_, CAT catalyzes the breakdown of hydrogen peroxide to water and molecular oxygen, and GPX decomposes peroxides [18]. 

Fish physical exercise can alter intracellular metabolism. Sustained or burst-type swimming can increase the demand for ATP and enhance O_2_ flow_,_ which are associated with high ROS production [19]. Swimming requires energy to move the large blocks of axial muscles arrayed along each side of the body, known as myotomes, which represent a dominant anatomical feature of most fishes. 

The aerobic red and anaerobic white muscle are the main types of striated muscle, and according to muscle type involved, three different types of swimming can be recognized: sustained, prolonged, and burst [20].

The burst-type swimming that is fueled by anaerobic glycolysis in white muscle can only be maintained for brief periods of less than 20 s and is terminated by exhaustion of intracellular energy supplies or by accumulation of waste products. By contrast, sustained swimming is fueled by aerobic metabolism carried out in the red muscle. In this case, fish can swim at lower speeds that they can sustain for hours, days, or even weeks. Between these two levels there is a zone of endurance swimming or prolonged exercise that can last between 2 and 200 min and, depending on the speed, is terminated by exhaustion. Hammer [21] assumes 2 BL (body length)/sec as a realistic figure for the endurance speed of many (average-sized) fish species.

Therefore, red muscle is the most suitable tissue for studying the existing relationship between aerobic fish metabolism and ROS production during sustained activity [22]. Indeed, red muscle adapts to endurance training in fish via improvement of the mitochondrial oxidative capacity through mitochondrial biogenesis and upregulation of antioxidant enzymes [23]. In this regard, Mortelette et al. [22] were the first to study oxidative metabolism in red muscle, correlating ROS production and swimming exercise in silver eel (*Anguilla anguilla*).

Accordingly, this study aimed at investigating the effects of dietary Tau on swimming performance in European seabass (*Dicentrarchus labrax*), by assessing both the critical swimming speed (U_crit_) and the metabolic oxygen consumption (MO_2_). With the aim to investigate the antioxidant effect of Tau supplementation in the diet, we also measured ROS production in fish blood by using the respiratory burst activity (RBA) assay. In parallel, the expression of a suite of genes coding for key antioxidant enzymes, such as SOD (superoxide dismutase), GPX (glutathione peroxidase), and CAT (catalase) was quantified in both red muscle and liver. 

The underling hypothesis for this study is that Tau could positively influence fish metabolism, and enhance swimming performance and antioxidant status. 

## 2. Materials and methods

### 2.1. Ethics Statement 

All procedures involving fish comply with the guidelines of the European Union Council (2010/63/EU) for the use of experimental animals and have been approved by a properly constituted Research Ethics Committee of the University of Insubria (CESA, Comitato Etico di Ateneo per la Sperimentazione Animale). Project identification Code #08/13 of November 7, 2013, signed by the President of the Ethics Committee, Prof. Elena Monti.

### 2.2. Fish Feeding Trial and Diets

The entire experiment was conducted at the recirculating aquaculture system (RAS) of the University of Insubria (Varese, Italy). Twenty-eight tagged sub-adult seabass with an initial mean body weight of 92.57 ± 20.33 g were divided equally into four circular experimental tanks of 600 L and let to acclimate for 7 days. During this period fish were fed daily with a commercial diet manufactured by Raanan Fish Feed Ltd.

The water parameters were monitored every day: temperature 20 ± 2 °C, pH 8.56, total N-NH_3_^-^ < 0.2 mg/L, N-NO_2_^-^ <0.02 mg/L, and salinity 22 g L^−1^. Dissolved oxygen was maintained at 99%–100% of the saturation value by adding pure O_2_ to the system.

After acclimation, and for all the duration of the experiment (64 days), fish were fed two isoenergetic diets (Control and 1.5% Tau) containing the same low percentage of FM and high percentage of SBM and with one of the diets being supplemented with 1.5% of Tau (Table 1). 

To prepare the diets, all the ingredients were ground and thoroughly mixed in a blender. Then, oil and a small quality of water were incorporated to make a smooth dough, which was extruded using an electric-extruder homemade pasta-machine with a mesh plate of 2 mm size. Extruded pellets were dried overnight at 45 °C and stored at −18 °C until further utilization. The formulation and the proximate composition of each diet are shown in Table 1. The Tau supplemented to one of the diets was organically produced at the Julita Farm (Julita, Sweden). Diets were given in duplicate (2 tanks/diet). Fish were fed every morning and at a feeding ratio of 1% body weight. 

Fish were individually weighed and measured for their length at the beginning of the experiment (t0), after 30 days of feeding (t1), and at the end of each test (t2, t3) (see below). These data were used to calculate weight gain (WG), specific growth ratio (SGR = 100 × (ln (final body weight) – ln (initial body weight))/days), feed conversion ratio (FCR = feed intake/WG), and condition factor (K = 100 × (wet weight/total length^3^)). Furthermore, in the interval between weightings, to avoid feeding restriction that may have limited the growth potential of fish, the daily feed portion was also adjusted based on European sea bass growth curves that are available at our lab. These curves have been generated with data obtained from previous growth trials conducted with the same fish species.

### 2.3. Fish Tagging

**Figure 1:**
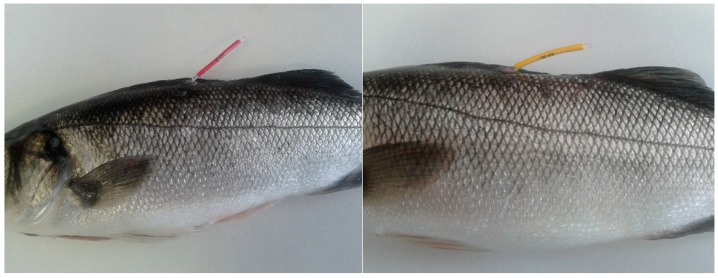
European seabass tagged with anchor tags of different colors and identification (ID) codes.

All fish were tagged by using streamer tags of different colors. These long, thin tags that stick out of the body of the fish are often referred to as anchor tags, or spaghetti tags, and have an identification code on them that we registered for each fish (Figure 1). 

Tags were placed under the dorsal pin of each fish and the anchor at their end was inserted into the dorsal muscle and locked into place between bones to hold the tag more securely. No tag loss or mortality was observed for the fish tagged in the study. The small wounds left by the needle used to implant the tags had completely healed in 2–3 days in all fish.

### 2.4. Swim Test and Sampling Intervals 

RBA, U_crit_, and MO_2_ were measured at different time-points during the feeding trial. The first RBA was measured at the beginning of the feeding trial (t_0_), whereas the second one was measured at 30 days of feeding (t_1_) with the experimental diets in both fish groups (Control and 1.5% Tau). For RBA analysis, blood was collected from five fish/diet. Fish were anesthetized and after blood collection (1 mL from each fish) they were placed to the same communal tank. 

After 4 days of recovery from blood collection and individual weight measurements the first swim stamina test was conducted (t2), measuring U_crit_, and MO_2_. Five fish/diet (not taken from the same tank) were tested for their swimming capacity for a total of 10 fish tested in five days (two fish per day were tested as just one swim-tunnel respirometer was used for the measurements). Fish were not fed the day of the swimming test. After the swimming test of each fish was finished, a blood sample was collected for RBA analysis and the fish was placed into the communal tank.

The second swimming stamina test (t_3_) was performed after 20 days of recovery from the first test (t2), following the same aforementioned protocol. U_crit_, MO_2_, and RBA were again measured from five fish/diet [24]. During the recovery time, fish were fed daily, whereas the day of the swim endurance test, fish were fasted. After the swimming exercise of each fish at t3 was concluded and a blood sample was collected for RBA analysis, fish were sacrificed and their tissues (liver and red muscle) were collected and used for gene expression analysis. The remaining fish in the communal tanks were anaesthetized in tricaine methanesulfonate (MS-222; 0.1 g/L, Sigma) and individually measured for their weight and length.

### 2.5. Fish Respiratory Metabolism and Swim Performance Measurements

An Auto^TM^ Respirometer (Loligo^®^ Systems, Viborg, Denmark) with a 10 L swim-chamber respirometer (10 cm × 10 cm × 40 cm) was employed to test fish in a non-turbulent water flow with a uniform velocity profile. A computerized system with fiber optic oxygen sensing technology, and the AutoResp™ software with the automated intermittent respirometry system allowed to measure oxygen uptake by fish. A flush pump provided water exchange between the respirometer and the buffer tank where the physio-chemical characteristics of the water were checked using a thermoregulator, an air pump, and a filter. Mean water temperature was maintained at 20.0 ± 0.1 °C and salinity at 22 g L^−1^. A data acquisition system continuously recorded oxygen saturation in the respirometer (mean oxygen saturation ± SEM (standard error of the mean) = 98.4 ± 0.4%).

To perform the swimming stamina test, fish were individually collected from their rearing tank using a knotless net and placed directly into the swimming chamber. We minimized fish capture time, avoided collecting multiple fish and did not hold fish for more than a few seconds in the net, as these factors can increase stress affecting swimming performance. Fish were left in the swim-tunnel respirometer to acclimate for 2 h in a current at a low speed of 0.7 BL (body length) s^−1^ (15 cm s^−1^). At this swimming velocity, the seabass was positioned on the bottom of the chamber, gently sculling its pectoral fins and occasionally the tail flicked. The chamber was covered with a black net to encourage fish to stay in the anterior part of it.

After the acclimation period, fish were exposed to gradual increments (every 30 min) of the swimming speed: 0.7, 1.4, 2.1, 2.8, 3.5, and then 4.2 BL s^−1^, until fatigue [25]. Fish were considered to be fatigued when they were unable to move themselves from the posterior screen of the swimming chamber despite gentle encouragement by sudden increases in current velocity [26]. At each speed, the swimming chamber was automatically set to complete three phases: 7 min of flushing, 1 min of waiting, and 7 min of measuring oxygen uptake levels. This kind of protocol is defined as intermittent stopped-flow respirometry [27]. The three phases were repeated twice in our experiment, thus the instantaneous oxygen uptake (MO_2_, in mg kg−1 h−1) was recorded every 15 min. 

To test recovery ability and determine individual variation in performance, the same fish were given a second swimming endurance test (t3) after a recovery period of 20 days in the communal tank. The same aforementioned protocol was followed for the second swimming test. 

The Ucrit values for both t2 (Ucrit1) and t3 (Ucrit2) swimming tests were calculated using the following formula [28,29]:

Ucrit = Ui+ (Uii (Ti/T ii))(1)

where

Ui is the highest velocity maintained for the entire interval (cm s^−1^);
Uii is the velocity increment (cm s^−1^);
Ti is the time elapsed at fatigue velocity (s), and Tii is the prescribed time interval (s).

The critical swimming speed values (Ucrit) for each fish were calculated in cm s^−1^ and converted in BL s^−1^. No correction for the solid blocking effect was considered, as the total cross-sectional area of the fish did not exceed 5% of the swimming chamber [30]. 

### 2.6. Respiratory Burst Activity 

The induction of respiratory burst activity (RBA) in blood leucocytes was measured according to the method described by Sitjà-Bobadilla et al. [31]. Briefly, 100 μL of diluted blood (1:25) in HBSS (Hank’s Balanced Salt Solution) (pH 7.4) was dispensed in white, flat-bottomed 96-wells, and incubated with 100 μL of a freshly prepared luminol suspension (2 mM luminol in 0.2 M borate buffer, pH 9.0) mixed with 1 μg ml^−1^ phorbol myristate acetate (PMA, Sigma) for 1 h at 24–25 °C. Luminol-amplified chemiluminescence was measured every 3 min using a plate luminescence reader to generate kinetic curves. Each sample was run twice and read against a blank to which neither blood nor PMA was added [32]. The integral luminescence in relative light units (RLU) was calculated.

### 2.7. Gene Expression Analysis

#### 2.7.1. RNA Extraction and cDNA Synthesis

Total RNA was extracted from 120 mg of liver and red muscle (5 fish/treatment). Lysis and homogenization of tissues were performed in special disposable sterile tubes (GentleMACS M tubes™, MiltenyiBiotec), in order to minimize the possibility of cross-contamination between samples and using the gentleMACS Dissociator (Miltenyi Biotec, Milan, Italy). The RNA was isolated through an automated purification method using Maxwell^®^ 16 Tissue LEV total RNA purification Kit and the Maxwell^®^ 16 Instrument (Promega, Milan, Italy). Concentration and purity of RNA were assessed by using the NanoDrop™ spectrophotometer Thermo Scientific™ (Thermo Fisher, Milan, Italy. RNA integrity was checked by electrophoresis on agarose gel (1%) stained with ethidium bromide. Then, 1 μg of RNA was transcribed into complementary DNA (cDNA) with GoScript™ Reverse Transcription System (Promega, Italy) using oligo d(T)16 primer. 

#### 2.7.2. Generation of Standard Curves for *cat*, *sod*, and *gpx* genes

The number of transcript copies of genes superoxide dismutase (*sod*, GenBank acc. no. FJ860004), catalase (*cat*, GenBank acc. no. FJ860003), and glutathione peroxidase (*gpx*, GenBank acc. no. FM013606) were quantified via One-Step Taqman^®^ real time PCR analysis with the standard curve method. 

Standard curves, one per each target gene, were constructed using the known copy number of a synthetic gene-specific mRNA. The entire protocol is described in detail in Terova et al. [33]. Briefly, a forward and a reverse primer were designed based on the coding sequence of each gene and used in a conventional RT-PCR to create templates for in vitro transcription. The nucleotide sequences of forward primers were engineered to contain a T7 promoter sequence at their 5′ end (Supplementary Table S1). PCR products were TA-cloned using the pGEM^®^-T easy vector system (Promega) and subsequently sequenced. 

In vitro transcriptions were performed using T7 RNA polymerase and the Promega RiboProbe In Vitro Transcription System kit, according to the manufacturer’s protocol. The concentration of the in vitro-transcribed RNAs was spectrophotometrically determined. Further details on the generation of standard curves are reported in Supplementary Material S1.

#### 2.7.3. One-Step TaqMan^®^ quantitative PCR Analysis 

One hundred nanograms of total RNA extracted from the biological samples were run in one-step qPCR, in parallel (in the same 96-well plate) to defined amounts of standard mRNAs (ten-fold serial dilutions), used to establish the standard curves. To reduce pipetting errors, master mixes were prepared to set up duplicate reactions (2 × 25 μL) for each sample. 

Real-time Assays-by-Design^SM^ PCR primers and gene-specific fluorogenic probes were manufactured by Thermo Fisher Scientific (Milan, Italy) (Supplementary Table S2). Raw data from qPCR runs were collected and analyzed with CFX Maestro™ Software (Bio-Rad, Milan, Italy). The Ct values were used to create standard curves to serve as a basis for calculating the absolute amounts of mRNA in total RNA extracted from each biological sample.

### 2.8. Statistical Analysis 

Statistics were performed using STATISTICA 7 software. Data related to the fish growth parameters, MO_2_, and U_crit_ were subjected to two-way ANOVA (Analysis of Variance) according to the GLM procedure, to determine differences due to the effect of time, diet, and their interaction (time x diet). 

Since the average initial weight of fish belonging to the two dietary groups was different, fish fed with Tau-fortified diet being heavier (although not significantly) than the control fish, a covariance analysis (ANCOVA) was performed to adjust subsequent fish weights and weight gains to a same initial weight.

The significance values of RBA at different times (t_0_, t_1_, t_2_, t_3_) were calculated with randomized block ANOVA, considering time as a block. 

An unpaired t-test was conducted to detect any differences in the expression of *cat, sod,* and *gpx* genes among the different diets. The level of significance was set at *p* < 0.05.

## 3. Results

### 3.1. Effect of Taurine on the Fish Performance Parameters and on the Metabolic Response

By considering time, diet, and their interaction as sources of variation, the increase in fish body weight (BW) from t1 through the end of the trial, showed statistical differences due to the factor diet (*p* < 0.01), being higher in fish fed a 1.5% Tau-supplemented diet than in the control group (Table 2). 

Similarly, the increase in BW over the trial was statistically significant for the factor time (*p* < 0.01), whereas the interaction between diet and time for BW did not result as statistically significant. SGR showed statistically differences for the factor diet (*p* < 0.01) being higher in fish fed the Tau-supplemented diet, whereas the interaction between diet and time for SGR did not result as statistically significant. No significant differences were reported for FCR and K between dietary groups and times (Table 2).

In Table 3, the MO_2_ values did not show any significant differences among the feeding groups due to diet and time.

At t_2_, fish fed with the 1.5 % Tau-supplemented diet showed an U_crit_ value significantly higher with respect to the control group (*p* < 0.01). Similarly, at t_3_ fish fed 1.5% Tau-supplemented diet recorded an U_crit_ value significantly higher than the U_crit_ value displayed by the control group (*p* < 0.01). Time determined a significant difference for the U_crit_ values recorded by the control and 1.5% Tau group at t_2_ and t_3_ (*p* < 0.01). The interaction between diet and time for U_crit_ did not result as statistically significant.

### 3.2. Respiratory Burst Activity

**Figure 2:**
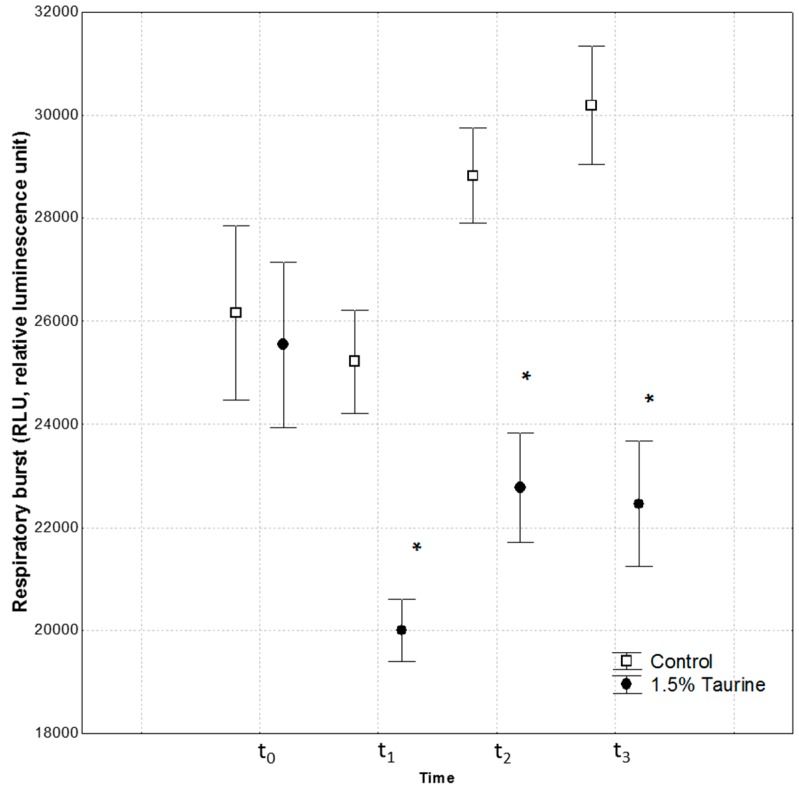
The RBA values after PMA stimulation in blood leucocytes of European seabass fed with control or with the 1.5% Tau-supplemented diet (mean ± SEM, n = 5 fish/diet). (*) means significant differences between dietary groups (*p* < 0.05) at t_1,_ t_2,_ and t_3_. No interaction between times (t) was detected.

Figure 2 shows the RBA in fish fed control and 1.5% Tau-supplemented diet at different sampling times. No statistically significant differences were detected between t2 and t3 sampling times for either group, so the duration of the recovery time was sufficient for fish. 

At t_1_, after 30 days of Tau administration, a significant difference in RBA levels was observed between seabass fed with the control diet and the group fed with the 1.5% Tau addition (*p* < 0.05). Subsequently, at t_2_ and t_3_, fish were subjected to the swim stamina test and the RBA assay was performed immediately thereafter in the blood. The RBA of leukocytes after PMA stimulation at t_2_ and t_3_ was significantly lower (*p* < 0.05) in fish fed with 1.5% Tau than in the group fed with the control diet. Indeed, seabass fed with 1.5% Tau consistently showed a tendency to produce less ROS than sea bass fed the control diet at each time point. Furthermore, in the control group, swimming performance increased the RBA from t_1_ to t_2_ and a further increase was recorded at t_3_.

### 3.3. Gene Expression Analysis

At the end of trial, the expression of genes involved in the oxidative stress response was evaluated by real-time PCR in fish liver and red muscle tissues. 

**Figure 3:**
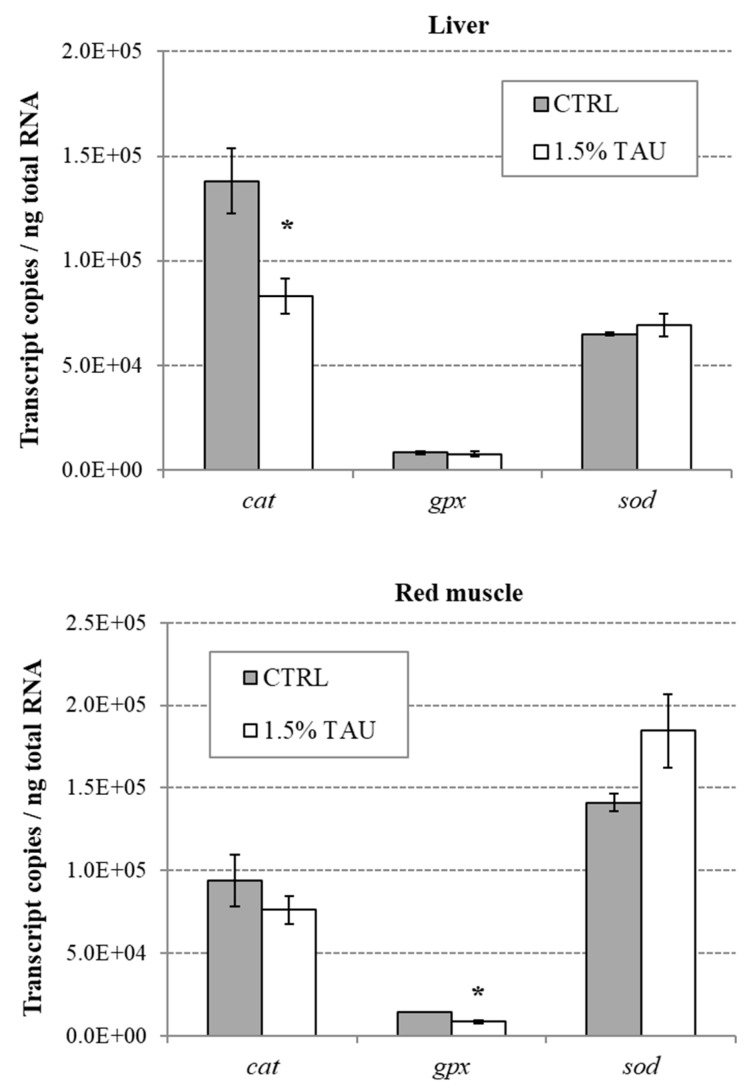
Absolute quantification of transcript copies of *cat, sod*, and *gpx* genes in liver and red muscle of European seabass fed with the control or with the 1.5% Tau-supplemented diet (mean ± SEM, n = 5 fish/diet). (*) means significant differences between dietary groups (*p* < 0.05).

Transcript copy number of the *cat* gene showed a significant down regulation in the liver of fish fed with the 1.5%Tau-supplemented diet in comparison to the group fed the control diet without Tau (*p* < 0.05; Figure 3). No significant differences in the number of *cat* mRNA copies were found in the red muscle tissue.

The *gpx* gene expression was significantly downregulated in the muscle of the 1.5% Tau dietary group with respect to the control (*p* < 0.05), as shown in Figure 3, whereas mRNA copies in the liver did not differ between the two feeding groups. 

In contrast, expression of the *sod* gene was not influenced by dietary Tau supplementation in either of the analyzed tissues (Figure 3; *p* > 0.05). 

## 4. Discussion

In aquaculture nutrition, one of the drawbacks of using plant-derived proteins to substitute FM in fish feed is related to the low level of Tau and indispensable amino acids (in particular, methionine and lysine). Indeed, Tau is almost lacking in plants with the exception of certain algae, seaweeds, and a few others, such as cactus pear. In fish, reduced growth rates and feed efficiency can be attributed to imbalances in the amino acidic profile of plant-derived proteins such as SBM. Jirsa et al. [34] showed a significant decrease in growth performance of white seabass (*Atractoscion nobilis*) fed with high levels of terrestrial plant-derived protein. Another study of the same research group, investigating the growth of the same fish species at the juvenile life stage, showed an improved performance due to 0.99% Tau supplementation in SBM-based diets [15]. In common dentex juveniles (*Dentex dentex*) sizing 39.1 g, a 40% replacement of FM with SBM decreased fish growth rates and feed efficiency, which were then improved by adding 2 g/kg of Tau in the diet [35]. The red sea bream (*Pagrus major*), meagre (*Argyrosomus regius*), and white seabream (*Diplodus sargus*) juveniles also benefited from diets low in FM containing 5–20 g/kg, 10 g/kg, and 1% Tau, respectively [10,36,37]. 

Martins et al. [38] showed that European sea bass (*D. labrax*) juveniles require a dietary Tau level of 0.47%–0.51% dry matter for maximum growth performance, whereas in the study of Brotons Martinez et al. [39], the SGR of seabass *(D. labrax)* fry was increased when 0.2% and 0.3% Tau was added to the feed, with respect to fry fed 0% and 0.1% Tau-supplemented diets. This suggests that the growth stimulatory effect of Tau supplementation in the diets is correlated with ontogenetic stages of fish [8,12]. In particular, larval and early juvenile developmental stages of marine fish are mostly influenced by dietary Tau (please see Reference [8] for a review), with the exception of gilthead seabream larvae in which Tau supplementation did not enhance growth performance [40]. This could suggest an ability of this fish species to synthetize Tau through the conversion of methionine into taurine.

For this reason, as reviewed by El-Sayed [8], juvenile stages could better show the beneficial effects of dietary Tau dietary supply than adult fish. Although our experimental design was not focused on growth performance, sub-adult seabass in our study showed higher body weight when fed on low-FM based diet supplemented with 1.5% Tau than on non-supplemented diet.

Tau is known for its role in protecting the organism from two major causes of cellular toxicity, oxidative stress and calcium overload [5]. However, the mechanism underlying the antioxidant activity of taurine is still not completely known [5]. In our experiment, the antioxidant action of Tau was studied through a swimming endurance test, which can generate excessive ROS, leading to oxidative stress-related impaired muscle contractility.

Seabass fed on 1.5% Tau showed a good swimming performance and U_crit_ was found to be significantly higher in the first swimming endurance trial (t_2_), with even more marked differences between the two dietary groups in the successive trial (t_3_). Indeed, adding Tau in the diet increased the swimming resistance in fish and the higher U_crit_ provided a maximum performance measure of sustained swimming not only in terms of speed but also of time elapsed at final velocity. A similar physical endurance enhanced by Tau was previously observed by Yatabe et al. [3] in rat fed a 0.5 g/kg dose of Tau. The authors also observed that oral Tau administration increased the concentration of Tau in the skeletal muscle. 

The increased U_crit_ observed in our experiment in fish fed with the 1.5% Tau-supplemented diet is in line with the results of Ripps and Shen [7] who reported that increasing taurine levels restores respiratory chain activity and increases the synthesis of ATP at the expense of superoxide anion production.

The link between swimming training and ROS production was studied for the first time by Mortelette et al. [22] in trained and untrained eels (A. anguilla). In trained eels, the red muscle fibers collected along the lateral line showed an increased ROS production (through hydroxyl radical, HO•, quantification) in vitro, as well as a higher maximal oxygen consumption than muscle fibers of untrained eel. The same result was previously described by Palstra et al. [41] on the same species, reporting an increase in oxygen consumption and ROS production in eel red muscle after forced swimming aerobic exercise. The results of the present in vivo study with European seabass are in agreement with both papers, confirming the results on ROS production associated with forced swimming. 

We also investigated ROS production by measuring the respiratory burst activity (RBA) in isolated leucocytes, at time t_2_ and t_3_ after swimming performance. Seabass fed on 1.5% Tau showed a reduction in RBA in comparison to the control group. Both swimming stamina tests (t_2_ and t_3_) induced an increase in ROS production in the control group, whereas dietary Tau in fish of the other group led to an increase in ROS but at much lower levels than in the control group. This result supports a role of Tau as a scavenger of ROS as described by Jong et al. [5], and Bañuelos-Vargas et al. [16]. In particular, Tau prevented the diversion of electrons from the respiratory chain to the acceptor oxygen forming in the process superoxide anion, by enhancing electron transport chain activity, and protecting the mitochondria against excessive superoxide generation [5]. Indeed, it is widely accepted that slowing the flux of electrons through the respiratory chain can divert electrons from complexes I-III to an alternate acceptor, such as oxygen [42]. Particularly, complexes I-III of respiratory chain are considered the primary mitochondrial sources for generating superoxide. In this regard, it has been documented that Tau deficiency compromises cellular activities [5]. 

With regard to oxygen consumption, no differences were detected between seabass dietary groups. The recovery time between t_2_ and t_3_ allowed fish to adapt their metabolism to swimming stamina test in both groups. 

Little is known about the relationship between ROS production and metabolic rate in ectotherms, especially in fish. Therefore, our study represents a contribution. Fish represent a good model to gain insights into the link between oxygen consumption and ROS production because variations of fish metabolic rate can be easily induced through experiments conducted under physiological conditions. In this regard, our study is in line with Mortelette et al. [43], who compared yellow and silver European eel (A. anguilla), and rainbow trout (O. mykiss), known to differ by their metabolic rates, oxygen consumption, and hydroxyl radical production in the mitochondria of red muscle fibers. The authors found that trout consumed more oxygen and produced more hydroxyl radical than silver eels, whereas yellow eel was in-between. This means that the more red muscle fibers consume oxygen, the higher the mitochondria activity is, and the higher ROS production is. The authors explained differences in oxygen consumption with differences in life condition by the species under study: the trout came from a fishery, the yellow eels (non-migrating stage) lived in a river, and the silver eels were prepared to migrate under extreme conditions [43]. 

In addition to the hydroxyl radical (HO^•^) parameter as a control of oxidative status, the proton leak was considered as an antioxidant mechanism by Skulachev [44] through uncoupling proteins (UCPs) at the mitochondrial level [43]. In trout cardiac muscle, it was estimated that 65% of oxygen consumption is due to proton leak [45]. At the mitochondrial level, proton leak through UCPs decreases the energy available from the electrochemical gradient, lowering ATP synthesis. So, the energy derived from the oxidation of metabolic fuels is dissipated and released as heat [46]. The oxidative forms of production may be even more attenuated by Tau, working as a buffer molecule. The buffering property of Tau is given by the pKa value of the amino group (9.0–8.6), contributing to the mitochondrial pH in the range of 7.5–8.5. Additionally, a proper Tau concentration is relevant for balancing the mitochondrial pH with the regulation of proton pumping due to proton leak, electron transport chain, and ATP-synthase. In fact, extracellular treatments with high Tau concentrations stabilized or buffered the mitochondrial functions, as observed in the protection against oxidative burst detected in reperfusion [6]. Considering the latter statement, adding 1.5% Tau in the diet of our fish mitigated ROS production, even under forced swimming conditions. This was probably due to buffer molecule property of managing the proton flux, protecting the mitochondrial integrity and directing protons for ATP production. 

A further support for Tau potent anti-oxidant properties, comes from the pattern of expression of genes coding for three antioxidant enzymes, analyzed in the present study. It is known that the antioxidant defense system includes enzymes such as SOD, GPx, and CAT that act as ROS scavengers protecting cell membrane lipids from oxidative damage [47]. A few reports exist concerning the effect of feed components on SOD, GPx, and CAT activity in fish [48,49] and no information exists about their gene expression levels following high oxidative-stress-inducing activity in marine fish fed with Tau-supplemented diets. 

In our study, Tau supplementation had a protective effect against oxidative stress induced by swimming endurance in seabass, as denoted by a significant decrease in *cat* and *gpx* expression in liver and muscle, respectively. The downregulation of *cat* and *gpx*, is in line with the other result found in the present study i.e., the reduction of ROS in seabass fed on 1.5% Tau in comparison to the control group. Indeed, high levels of antioxidant enzymes demonstrate a stress-induced adaptive response in attempting to neutralize the generated ROS [50] and one of the benefits of taurine is that it likely decreases the production of ROS in the first place. 

## 5. Conclusions

The feed additive Tau was beneficial in low FM-based diets for European seabass, as it enhanced fish growth performance and decreased ROS production under conditions of oxidative-stress-inducing activity, such as forced swimming. The protective effect of Tau against oxidative stress was denoted also by a significant decrease in antioxidant enzymes gene expression in fish liver and muscle, confirming the role of Tau as an ROS scavenger in these tissues.

## Figures and Tables

**Table 1:** Ingredients (%) and proximate composition (%) of experimental diets.

Raw Material (%)	Diets	
	Control	1.5 % Tau
Fish meal	10.0	10.0
Full fat soy	12.8	12.8
SPC	13.6	13.6
Wheat	8.0	8.0
Wheat gluten meal	8.19	8.19
DCP	1.72	1.72
Mixed oil ^a^	12.0	12.0
Lysine	0.29	0.29
Vitamins and minerals premix ^b^	0.4	0.4
Corn gluten	16.0	14.5
Soybean meal	16.7	16.7
Anti moulds	0.1	0.1
Taurine	-	1.5
Proximate composition (%)		
Crude protein	45.0	45.0
Fat	16.0	16.0
Fiber	2.3	2.3
Ash	6.4	6.4
Calcium	1.0	1.0
Phosphorus	0.95	0.95
Methionine	0.9	0.9
Methionine+cysteine	1.6	1.6
Lysine	2.3	2.3

SPC soy protein concentrate, DCP dicalcium phosphate. ^a^ Mixed oil: 40% corn, 60% soy; ^b^ Vitamin and mineral premix (quantities in 1 kg of mix): vitamin A, 4,000,000 IU; vitamin D3, 800,000IU; vitamin C, 25,000 mg; vitamin E, 15,000 mg; inositol, 15,000 mg; niacin, 12,000 mg; choline chloride, 6000 mg; calcium pantothenate, 3000 mg; vitamin B1, 2000 mg; vitamin B3, 2000 mg; vitamin B6, 1800 mg; biotin, 100 mg; manganese, 9000 mg; zinc, 8000 mg; iron, 7000 mg; copper, 1400 mg; cobalt, 160 mg; iodine 120 mg; anticaking and antioxidant + carrier, making up to 1000 g.

**Table 2:** Body weight, specific growth rate, feed conversion ratio, and condition factor values of European seabass fed with control or 1.5% Taurine supplementation diet.

Time	t_0_		t_1_		t_2_		t_3_			*p*-Value		
Diet	Control	1.5% Tau	Control	1.5% Tau	Control	1.5% Tau	Control	1.5% Tau	RMSE	Time	Diet	Time x Diet
**BW (g)**	86.43	93.71	102.64	116.43	104.14	122.00	108.38	130.60	23.85	<0.01	<0.01	0.973
**SGR (%/day)**	-	-	1.13	1.17	0.78	0.86	0.68	1.17	0.99	0.25	0.05	0.09
**FCR**	-	-	1.08	1.10	1.11	1.14	1.15	1.18	0.16	0.47	0.63	0.44
**K (%)**	1.51	1.43	1.65	1.74	1.82	1.66	1.36	1.62	0.21	0.06	0.41	0.46

BW = Body weight; SGR = specific growth rate; FCR = feed conversion ratio; K = condition factor; Tau = Taurine; RMSE = root mean square error.

**Table 3:** Effects of diet on metabolic responses to exercise and critical swimming speed performance at t_2_ and t_3_ in the European seabass.

Time	t_2_		t_3_			*p*-Value		
Diet	Control	1.5% Tau	Control	1.5% Tau	RMSE	Time	Diet	Time x Diet
MO_2_	271.50	266.20	289.0	272.80	52.40	0.94	0.26	0.38
U_crit_	3.97	4.16	4.27	4.60	0.23	<0.01	<0.01	0.31

**M**O_2_ = metabolic oxygen consumption; U_crit_ = critical swimming speed; RMSE = root mean square error.

## Supplementary Materials

The following are available online at https://www.mdpi.com/2076-2615/9/9/607/s1.
