# Supplementary material for: Protective Effect of Dietary Taurine from ROS Production in European Seabass under Conditions of Forced Swimming
Source: Animals (Basel). 2019 Aug 26;9(9):607. doi: 10.3390/ani9090607 (PMC6770007; doi:10.3390/ani9090607)
Supplement: Supplementary file 1 [file animals-09-00607-s001.pdf]

animals

PROTECTIVE EFFECT OF DIETARY TAURINE FROM ROS PRODUCTION IN EUROPEAN SEABASS UNDER CONDITIONS OF FORCED SWIMMING.

Chiara Ceccotti[1], Basim S.A. Al-Sulaivany[2], Omar A.M. Al-Habbib[3], Marco Saroglia[1], Simona Rimoldi[1], and Genciana Terova[1,*]

[1] Department of Biotechnology and Life Sciences, University of Insubria, 21100 Varese, Italy
[2] Department of Biology, Faculty of Science, University of Zakho, Zakho, 420011 Kurdistan Region-Iraq
[3] College of Science, International University of Erbil, 44001 Kurdistan Region-Iraq
* Correspondence: genciana.terova@uninsubria.it, Tel.: +39 0332 421 428

Supplementary material S1

Generation of standard curves for cat, sod, and gpx genes

To generate the standard curves defined amounts of mRNAs at tenfold dilutions were subjected to qPCR using iTaqTM Universal Probes One-Step Kit (Bio-Rad, Italy) and Bio-Rad® CFX96TM Real-Time PCR System. The thermal cycling protocol was 10 min at 50 °C, 3 min at 95 °C, followed by 40 cycles consisting of 15 s at 95 °C, and 1 min at 60 °C. The cycle threshold (Ct) values obtained by amplification were used to create standard curves for target genes. This curve served as a basis for calculating the unknown mRNA copies of each gene in each liver and muscle RNA sample.

Table S1 *Sequences of primers used to synthesize standard RNA with relative accession number.*

	Symbol	Acc. nr.	Primer Sequence (5'-3')
Superoxide dismutase	sod	FJ860004	F:gtaatacgactcactatagggGTTGGAGACCTGGGAGATGT<br>R:GAAAAGGAGGCAATGAGGAG
Catalase	cat	FJ860003	F:gtaatacgactcactatagggATGGTGTGGGACTTCTGGAG<br>R: CGTTTCTACTGCAAGTTCCACT
Glutathione peroxidase	gpx	FM01366	F:gtaatacgactcactatagggAGTTAATCCGGAATTCGTGAGA<br>R: CAACAACCAGGGACTACACTCA

Table S2 *Primer sequences and TaqMan® probe of each target gene.*

Gene	*Symbol*	*Nucleotide sequence (5′-3′)*
Superoxide Dismutase	*sod*	*F: TGGAGACCTGGGAGATGTAACTG*<br>*R: CAAGATAGACATCACGGACAAGA*<br>*Taqman Probe: CAGGAGGAGATAACATTG*
Catalase	*cat*	*F: ATGGTGTGGGACTTCTGGAG*<br>*R: CATCAGGTGTCTTTCTTGTTCAGC*<br>*Taqman probe: TGAGGCCTGAGTGTCTG*
Glutathione Peroxidase	*gpx*	*F: AGTTAATCCGGAATTCGTGAG*<br>*R:GTTTTACGACCTGACAGCTAAGCT*<br>*Taqman probe: AATGGCTGGAAACGTG*
